# Subthalamic nucleus long-range synchronization—an independent hallmark of human Parkinson's disease

**DOI:** 10.3389/fnsys.2013.00079

**Published:** 2013-11-19

**Authors:** Shay Moshel, Reuben R. Shamir, Aeyal Raz, Fernando R. de Noriega, Renana Eitan, Hagai Bergman, Zvi Israel

**Affiliations:** ^1^Department of Medical Neurobiology, IMRIC, The Hebrew University-Hadassah Medical SchoolJerusalem, Israel; ^2^The Interdisciplinary Center for Neural Computation, The Hebrew UniversityJerusalem, Israel; ^3^The Edmond and Lily Safra Center for Brain Sciences, The Hebrew UniversityJerusalem, Israel; ^4^The Research Laboratory of Brain Imaging and Stimulation, The Jerusalem Mental Health Center, Kfar-Shaul Etanim, Hebrew University-Hadassah Medical SchoolJerusalem, Israel; ^5^Department of Anesthesiology, University of Wisconsin School of Medicine and Public HealthMadison, WI, USA; ^6^Department of Neurosurgery, Center for Functional and Restorative Neurosurgery, Hadassah University HospitalJerusalem, Israel; ^7^Department of Psychiatry, Hadassah University HospitalJerusalem, Israel

**Keywords:** Parkinson's disease, subthalamic nucleus, deep brain stimulation, oscillations, synchronization

## Abstract

Beta-band synchronous oscillations in the dorsolateral region of the subthalamic nucleus (STN) of human patients with Parkinson's disease (PD) have been frequently reported. However, the correlation between STN oscillations and synchronization has not been thoroughly explored. The simultaneous recordings of 2390 multi-unit pairs recorded by two parallel microelectrodes (separated by fixed distance of 2 mm, *n* = 72 trajectories with two electrode tracks >4 mm STN span) in 57 PD patients undergoing STN deep brain stimulation surgery were analyzed. Automatic procedures were utilized to divide the STN into dorsolateral oscillatory and ventromedial non-oscillatory regions, and to quantify the intensity of STN oscillations and synchronicity. Finally, the synchronicity of simultaneously vs. non-simultaneously recorded pairs were compared using a shuffling procedure. Synchronization was observed predominately in the beta range and only between multi-unit pairs in the dorsolateral oscillatory region (*n* = 615). In paired recordings between sites in the dorsolateral and ventromedial (*n* = 548) and ventromedial-ventromedial region pairs (*n* = 1227), no synchronization was observed. Oscillation and synchronicity intensity decline along the STN dorsolateral-ventromedial axis suggesting a fuzzy border between the STN regions. Synchronization strength was significantly correlated to the oscillation power, but synchronization was no longer observed following shuffling. We conclude that STN long-range beta oscillatory synchronization is due to increased neuronal coupling in the Parkinsonian brain and does not merely reflect the outcome of oscillations at similar frequency. The neural synchronization in the dorsolateral (probably the motor domain) STN probably augments the pathological changes in firing rate and patterns of subthalamic neurons in PD patients.

## Introduction

The subthalamic nucleus (STN) plays a critical role in the control of basal ganglia activity (Kitai and Kita, 1987; Nambu et al., 2002). In Parkinson's disease (PD), midbrain dopaminergic neurons degenerate, leading to a cascade of physiological changes that strongly affect the STN (Bergman et al., 1994; Hamani et al., 2004). Inactivation (Bergman et al., 1990; Aziz et al., 1991; Alvarez et al., 2009) and deep brain stimulation (DBS, Benazzouz et al., 1993; Pollak et al., 1993; Benabid et al., 1994; Weaver et al., 2009; Follett et al., 2010; Moro et al., 2010; Williams et al., 2010; Bronstein et al., 2011; Lhommée et al., 2012; Odekerken et al., 2013; Schuepbach et al., 2013) of the STN are highly effective in the management of advanced PD.

Neuronal oscillations, at the level of action-potential (spike) discharge (Rodriguez-Oroz et al., 2001; Kuhn et al., 2005; Moran et al., 2008; Zaidel et al., 2010; Guo et al., 2012; Lourens et al., 2013) and local field potential (Kuhn et al., 2009; Chen et al., 2010; Giannicola et al., 2010; Rosa et al., 2011) have been observed in physiological studies of the STN of PD patients undergoing DBS surgery. LFPs span the frequency range of 1–70 Hz [or 1–400 Hz, if one include the high gamma peaks reported at 65–90 Hz and 250–350 Hz (Danish et al., 2007), but see (Yuval-Greenberg et al., 2008) for possible confounding factors in the high frequency regime of LFP], whereas spikes have their maximal power around 1000 Hz. Thus, although LFP oscillations have been thought to imply spike synchronization (Brown and Williams, 2005; Hammond et al., 2007; de-Solages et al., 2011), they more likely represent sub-threshold phenomena such as synaptic activity (Belitski et al., 2010; Buzsaki et al., 2012) which is probably correlated with spike activity.

Conclusive evidence of the correlation (and causality) between neuronal oscillations and synchronization in the PD STN has remained elusive. Physiological studies of neuronal synchronization in the STN of the MPTP primate model are not yet reported. Robust oscillatory synchronization patterns of STN spiking activity have been reported in the 6-hydroxydopamine rodent model of Parkinsonism (Machado et al., 2006; Mallet et al., 2008a,b; Lintas et al., 2012). In human PD patients, oscillatory synchronization of spiking activity has been reported in several studies (Levy et al., 2000, 2002a,b; Amirnovin et al., 2004; Weinberger et al., 2006; Hanson et al., 2012; Alavi et al., 2013; Lourens et al., 2013) but there have been no detailed descriptions of the dependence of the neuronal synchronization on the oscillatory activity or the spatial properties of the neuronal pairs (e.g., simultaneous recording of neurons from the oscillatory and non-oscillatory regions of the STN, see below).

Previous studies have shown that the STN of PD patients can be divided into a dorso-lateral oscillatory region (DLOR) and ventro-medial non-oscillatory region (VMNR) (Moran et al., 2008; Zaidel et al., 2010; Seifried et al., 2012; Guo et al., 2013). The first aim of this study was to explore the properties of neuronal (spike) synchronization of the STN of human PD patients, principally within and between the different STN domains. The second goal was to further explore the relationship between oscillations and synchronization phenomena in the neural activity of the STN.

To overcome the inherent technical difficulties of spike isolation (Joshua et al., 2007; Hill et al., 2011) and spike sorting (Lewicki, 1998) in the electrically noisy environment of the human operating room, and to increase the sensitivity of correlation analysis (Bedenbaugh and Gerstein, 1997; Gerstein, 2000) this study used the unresolved collective (multi-unit) spiking activity recorded by two different microelectrodes exploring the boundaries and the domains of the STN during DBS procedures. This enabled the exploration of the properties of long-range correlation in the STN, in contrast to correlation studies of the activity recorded by a single electrode (e.g., Moran et al., 2008) which can only reveal short range correlations.

## Materials and methods

### Patients and surgery

Simultaneous microelectrode recordings from two electrodes in patients with Parkinson's disease (PD) undergoing surgery for subthalamic nucleus (STN) deep brain stimulation (DBS) were analyzed in this study. All patients met accepted criteria for STN DBS and signed informed consent for surgery. Microelectrode recording is performed to accurately localize STN borders and domains, in order to optimize the placement of the DBS electrode and thus enhance the therapeutic effects of the DBS procedure. The data collection was therefore done as part of our routine procedures and not part of a clinical trial. This study was authorized and approved by the Institutional Review Board of Hadassah University Hospital in accordance with the Helsinki Declaration (reference codes: 0545-08-HMO and HMO: 10-18.01.08).

Surgery was performed using a CRW stereotactic frame (Radionics, Burlington, MA, USA). STN target coordinates were chosen as a composite of the indirect anterior commissure-posterior commissure (AC-PC) atlas- based location and direct (1.5 or 3 Tesla) T2 magnetic resonance imaging (MRI), using Framelink 4 or 5 software (Medtronic, Minneapolis, USA). The recordings used in this study were made while the patients were awake without sedation. The patient's level of awareness was continuously assessed clinically and, if drowsy, the patient was stimulated and awoken through conversation by a member of the surgical team. Data were obtained while the patients were off dopaminergic medication, which was stopped 12 h prior to surgery.

### Microelectrode recordings

Data were acquired with the MicroGuide system (Alpha-Omega Engineering, Nazareth, Israel). Neurophysiological activity was recorded using polyamide coated tungsten microelectrodes (Alpha Omega) with impedance mean ± standard deviation (*SD*) of 0.60 ± 0.11 MΩ (measured at 1 kHz at the beginning of each trajectory). The signal was amplified by 10,000, band-passed filtered from 250 to 6000 Hz using four-pole Butterworth filter hardware, and sampled at 48 kHz by a 12-bit A/D converter (using ±5 V input range). Local field potentials were not recorded due to constraints of electrical noise in the operating room.

Microelectrode recording was performed using two parallel microelectrodes starting 10 mm above the estimated center of the dorsolateral STN target, based on the pre-operative T2 MRI image. The two electrodes were simultaneously advanced, and therefore the distance between the two electrodes was fixed (2 mm) during all recordings. Trajectories followed a double-oblique approach (approximately 60° from the axial AC-PC plane and 15° from the mid-sagittal plane) toward the STN target. The angles of the trajectory were slightly modified to avoid the cortical sulci, the ventricles and major blood vessels as revealed by gadolinium-enhanced T1 MRI (Machado et al., 2006). The “central” electrode was directed at the center of the STN target, and an “anterior” (ventral) electrode was located 2 mm anterior to the central electrode. Typically, the electrodes were advanced in steps of ~100μm between successive recordings sites within the STN. Only trajectories where both electrodes had passed through the STN for at least 4 mm were used in this study (yielding 72 trajectories of 2 electrodes from 57 PD patients undergoing bilateral STN deep brain stimulation surgery). After identification of the STN ventral border by the electro-physiologist, the STN and its sub- regions were automatically detected using the Hidden Markov model (HMM) method (Zaidel et al., 2009).

### Database

We studied 72 STN trajectories (each of 2 electrodes) from 57 PD patients, 40 males and 17 females, aged 58.9 ± 10.3 years (mean ± standard deviation, *SD*) and with disease duration of 10.3 ± 4.7 years (mean ± *SD*). The UPDRS motor part score, UPDRS III, was 49.2 ± 17.8 (mean ± *SD*) when assessed off dopamine replacement therapy before surgery. Patient details and clinical effects of the surgery are given at Table 1.

**Table 1:** **Summary of clinical data, before and after the DBS surgery, include medical treatment [total daily levodopa equivalent dose (LED)] and the motor part of patients rate scale for Parkinson; The Unified Parkinson's Disease Rating Scale, part 3 (UPDRS III; maximum value is equal to 108)**.

**Patient number**	**Age (years)**	**Disease duration (years)**	**Gender**	**UPDRS III**							**Medications (LED) daily doses**		
				**Before DBS (Med: On\Off)**		**After DBS: (Stim\Med: On\Off)**							
				**On**	**Off**	**On, On**	**On, Off**	**Off, On**	**Off, Off**	**Months since DBS**	**Before STN DBS**	**After STN DBS**	**Months Since DBS**
1	62	8	M	10	39.5	21	23	34	34	12	400	1100	8
2	73	10	M	23	58.5	NA	NA	NA	NA	NA	675	200	NA
3	51	14	F	17	99	2	16	9	46	13	1700	875	NA
4	68	9	M	NA	NA	NA	NA	NA	NA	NA	1662.5	1246.9	6
5	31	13	F	19	62	14	11		53	12	NA	0	NA
6	58	22	M	22.5	54	5	14	61	64	8	2575	900	8
7	73	6	M	22	55	5	18	NA	NA	4	1700	700	NA
8	75	8	F	17	51	17	21	24	37	8	1400.1	897.75	7
9	61	5	F	38	73	1	10		47	8	1250	125	5
10	56	8	M	29	70	NA	NA	NA	NA	NA	875	250	NA
11	63	12	F	19	49	5	16			3	890	459	NA
12	49	10	M	NA	NA	NA	NA	NA	NA	NA	900	NA	NA
13	64	11	F	3	21	2	4	7	21	5	1505	437.5	4
14	61	6	M	32	60	10	14	51	61	3	700	200	2
15	39	10	F	16	47	5	18	27	57	5	512.5	307.5	3
16	61	11	M	10	44	NA	NA	NA	NA	NA	500	510	NA
17	73	20	M	16	60	NA	NA	NA	NA	NA	1496	NA	NA
18	56	13	F	NA	NA	NA	NA	NA	NA	NA	NA	NA	NA
19	62	17	M	22	72	NA	NA	NA	NA	NA	1550	250	NA
20	70	13	F	13	41	NA	NA	NA	NA	NA	400	NA	NA
21	53	9	M	10	41	NA	NA	NA	NA	NA	375	NA	NA
22	72	8	M	4	17	NA	NA	NA	NA	NA	1125	NA	NA
23	50	8	F	NA	NA	NA	NA	NA	NA	NA	550	NA	NA
24	62	5	M	NA	NA	NA	NA	NA	NA	NA	NA	NA	NA
25	58	7	M	14	42	NA	NA	NA	NA	NA	300	NA	NA
26	63	12	M	19	55	NA	NA	NA	NA	NA	1105	NA	NA
27	52	5	M	3	26	NA	NA	NA	NA	NA	650	NA	NA
28	53	10	F	9	42	NA	NA	NA	NA	NA	770	NA	NA
29	74	9	F	42	73	NA	NA	NA	NA	NA	1250	NA	NA
30	57	7	M	37	78	NA	NA	NA	NA	NA	1370	NA	NA
31	67	9	M	14	34	NA	NA	NA	NA	NA	1000	NA	NA
32	59	10	M	13	47	NA	NA	NA	NA	NA	NA	NA	NA
33	55	8	M	22	69	NA	NA	NA	NA	NA	1480	NA	NA
34	53	8	M	32	49	NA	NA	NA	NA	NA	750	NA	NA
35	51	NA	M	29	43	NA	NA	NA	NA	NA	562.5	NA	NA
36	61	5	M	0	0	NA	NA	NA	NA	NA	NA	NA	NA
37	53	3	M	6	49	NA	NA	NA	NA	NA	200	NA	NA
38	NA	4	M	34	56	NA	NA	NA	NA	NA	850	NA	NA
39	74	15	M	31	57	NA	NA	NA	NA	NA	750	NA	NA
40	71	22	M	35	51	NA	NA	NA	NA	NA	2000	NA	NA
41	66	3.5	F	16	46	NA	NA	NA	NA	NA	750	NA	NA
42	66	10	M	11	63	NA	NA	NA	NA	NA	600	NA	NA
43	71	NA	F	6.5	28.5	15	23	56	59	28	1525	100	NA
44	51	12	M	NA	NA	4	3	48	31	23	1025	731.5	10
45	46	NA	M	1	25.5	NA	NA	NA	NA	NA	900	NA	NA
46	60	11	M	10.5	42.5	NA	NA	NA	NA	NA	1064	399	24
47	59	6	M	15.5	40.5	23	29	40	38	20	500	250	NA
48	56	10	M	6.5	37.5	12	20	23	31	26	900	675	NA
49	72	16	M	NA	NA	NA	NA	NA	NA	NA	1500	200	23
50	54	5	F	11	43.5	10	14	NA	60	22	850	600	NA
51	55	NA	M	7.5	36.5	NA	NA	NA	NA	NA	1150	450	NA
52	47	8	M	7.5	51	NA	NA	NA	NA	NA	1200	722.12	NA
53	42	9	F	4.5	43	NA	NA	NA	NA	NA	187	250	NA
54	29	9	M	NA	NA	NA	NA	NA	NA	NA	1200	612.5	NA
55	69	12	M	7.5	28.5	12	13	27	21	14	1609.5	768.13	NA
56	53	20	M	39.5	83	NA	NA	NA	NA	NA	2200	537.5	NA
57	61	22	F	15.5	56.5	NA	NA	NA	NA	NA	1000	500	18
Mean	58.9	10.3	M40/F17	17.2	49.2	9.58	15.7	34	44	12.588	1037.3	508.48	9.83
STD	10.3	4.7		11.1	17.8	6.75	6.65	18	15	8.3221	516.56	309.06	7.6

Mean and STD are respectively for the mean and standard deviation of each column values.

The minimal recording time duration of a STN pair to be included in this study was 5 s (analysis of the subset of recording with minimal recording duration of 10 s reveal similar results, data not shown). A total of 2390 multi-unit pair sites, in which both electrodes were judged to be inside the STN for the minimal duration, were studied. The same data base was used for the single site (oscillation) analysis, yielding 4780 single STN sites. Recording (and analysis) time duration of the STN pairs equaled 23.7 ± 25.3 s (mean ± *SD*).

### Analysis of synchronization and oscillations

All data analysis utilized custom-made MATLAB 7.10b (R2010.b) routines. The local field potential frequency domain was filtered out by the recording apparatus. Burst frequencies below the range of the operating room band-pass filter (250–6000 Hz) could be detected using the rectified signal, which follows the envelope of multi-unit activity (Moran et al., 2008; Halliday and Farmer, 2010; Moran and Bar-Gad, 2010; Zaidel et al., 2010). The raw 250–6000 Hz analog signal was therefore rectified by the “absolute” operator and the global mean was subtracted. Thus, the resulting analysis represents only spike activity.

The average power spectrum density (PSD) at each site was calculated using Welch's method with a 1.5 s Hamming window (50% overlap), after removing the local window mean, and with a 131,072 FFT points (nfft), yielding spectral resolution of 1/3 Hz [nfft = 2^∧^round(log2(Fs/f_res)), where *F*s = sampling frequency and f_res is the spectral resolution]. PSD amplitude is affected by the amplitude of the recorded neural activity, which is impacted by non-physiological factors such as the impedance of the electrode (Zaidel et al., 2010). To create homogenous PSD results for all recorded sites, the “relative” (normalized) power spectral density was calculated by dividing it by the total power of the signal between 0 and 3000 Hz. This relative, or normalized, power spectral density therefore estimates the spectral peak in relation to the other peaks in the spectrogram.

To compute coherence, the magnitude squared (MS-) coherence method (Kay, 1988; Miller and Sigvardt, 1998) was used. Welch's method was utilized, with a 1.5 s Hamming window (50% overlap), after removing the local window mean and with a spectral resolution of 1/3 Hz (same conventions as for PSD). Coherence values are limited (by definition) between 0 and 1. All coherence averages were therefore calculated in Fisher's transform domain (Miranda de Sa et al., 2009) and then reversed.

By definition, the removal of each window mean in the spectrum and the coherence analysis eliminate any power at 0 Hz (DC). We therefore start all the spectrum and coherence plot of this manuscript at 1 Hz.

A constant baseline level emerged in our coherence results (e.g., Figures 4A,B). This baseline probably resulted from the finite sampling of two “random noise” sources. To verify this, pairs of Gaussian random noise sources were simulated. The simulated data were subjected to the same filters and absolute operator as the real neuronal data and the same analysis tools. The magnitude of the coherence baseline dropped exponentially as time duration increased. Therefore, the baseline level seen in the STN coherence is most likely due to the finite (and relatively) short duration (mean = 23.7 s) of the recordings in human patients. The coherence functions were normalized by the subtraction of the average coherence of the randomly shuffled (10,000 times) pairs from the same STN domain. Note that the normalized coherence functions can therefore display negative coherence values.

### Synchronization and oscillation strength (*sync* and *oscil scores*)

**Figure 1:**
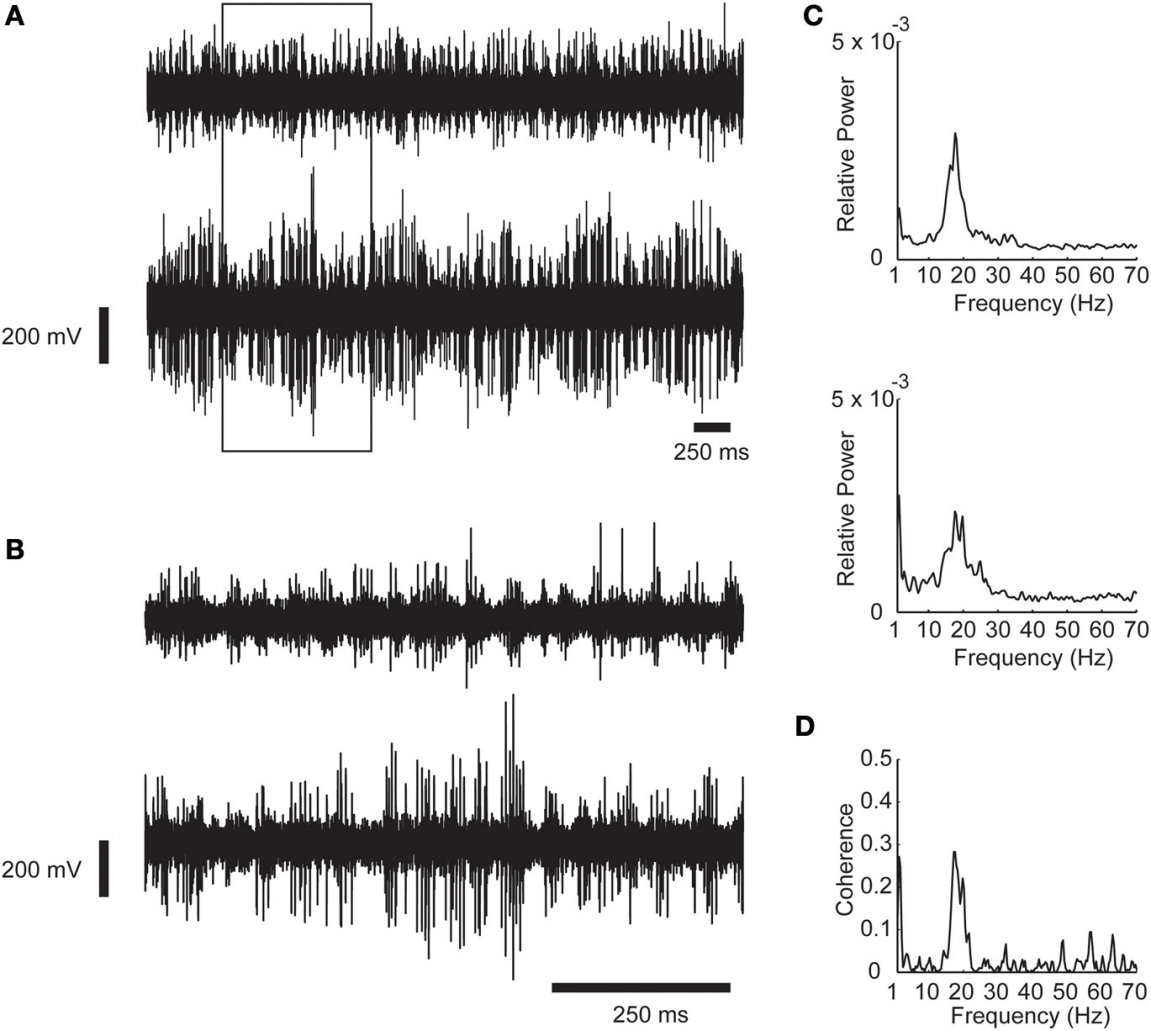
**An example of synchronous oscillations between two recording sites in the STN. (A)** Raw analog data. Data is recorded by two microelectrodes separated horizontally by a distance of 2 mm, hardware band pass filtered between 250 and 6000 Hz, and digitally sampled at 48 KHz. The 1 s data in the rectangle is expanded in **(B)**. **(C)** Power spectrum of the recording shown in **(A)**. **(D)** Coherence function of the two STN sites. Oscillations scores = 31.57 and 17.13 respectively; Synchronization score = 4.89.

**Figure 2:**
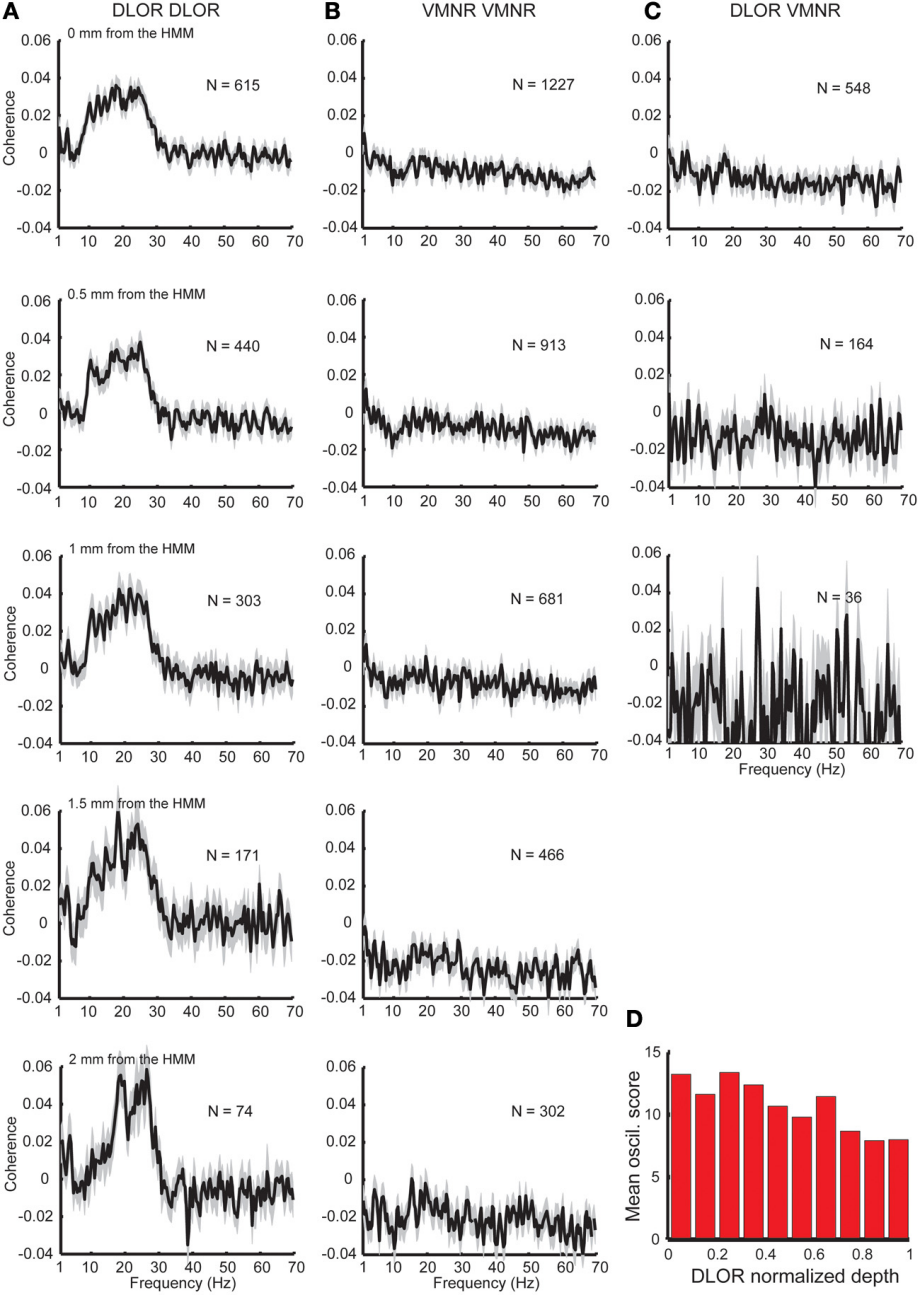
**Synchronization found only between sites in the DLOR. (A)** Substantial coherence at beta frequency range exists between sites within the DLOR (from the STN entrance to the DLOR\VMNR border). However, the relative coherence is around zero in the beta range for pairs recording from the VMNR **(B)**, or between sites of the DLOR and VMNR **(C)**. HMM was used to automatically define the DLOR-VMNR border. The first row is calculated using the HMM defined border, and lower rows calculated with a progressively increasing gap from the HMM border. *N* is the number of pairs, and the shaded areas represent the standard error of the mean (SEM) of the relative coherence function for each population. **(D)** Distribution of oscillations scores along the normalized DLOR depth. For each trajectory the DLOR length was normalized for 0-entry, 1–end of DLOR.

**Figure 3:**
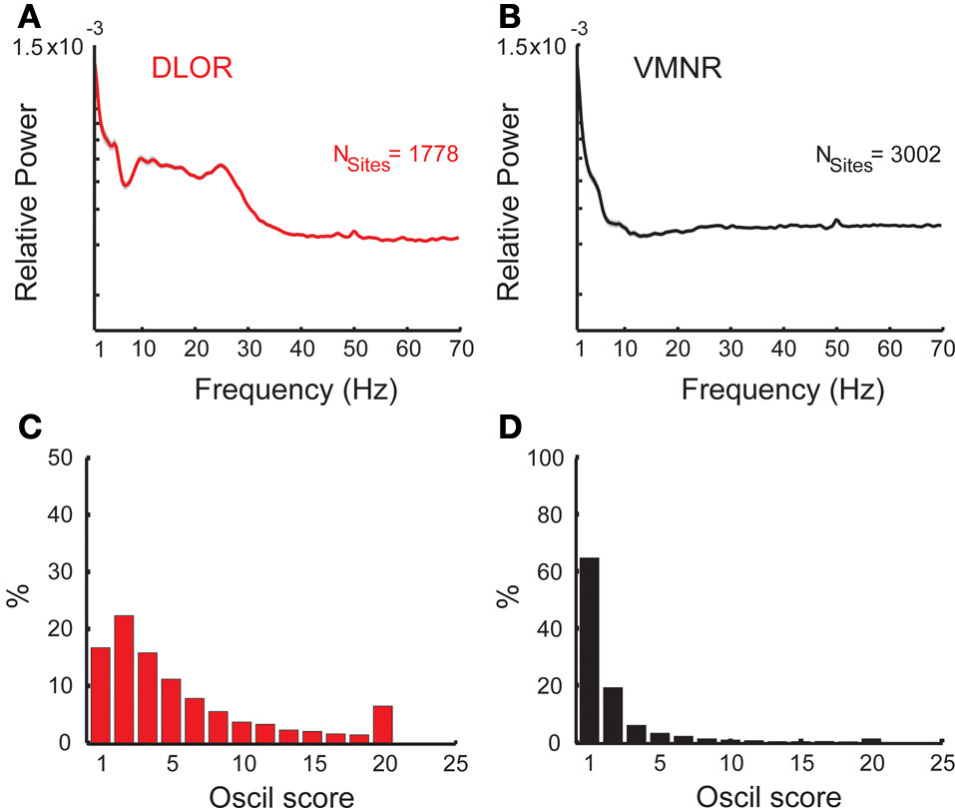
**Oscillatory activity in the STN DLOR and VMNR.** Average power spectra of STN activity of DLOR **(A)** and VMNR **(B)** recordings. The shaded areas represent the standard error of the mean (SEM) of the spectrum function for each population. *N*_sites_ is the number of sites averaged. **(C,D)** Distribution of the *oscil scores* in the DLOR and the VMNR recordings. Note the different scales of the *Y*-axes. *Oscil scores* below zero and above 20 (*n* = 68 and 153 out 4780) are appended to the first and last bin respectively to enhance visualization of the results.

**Figure 4:**
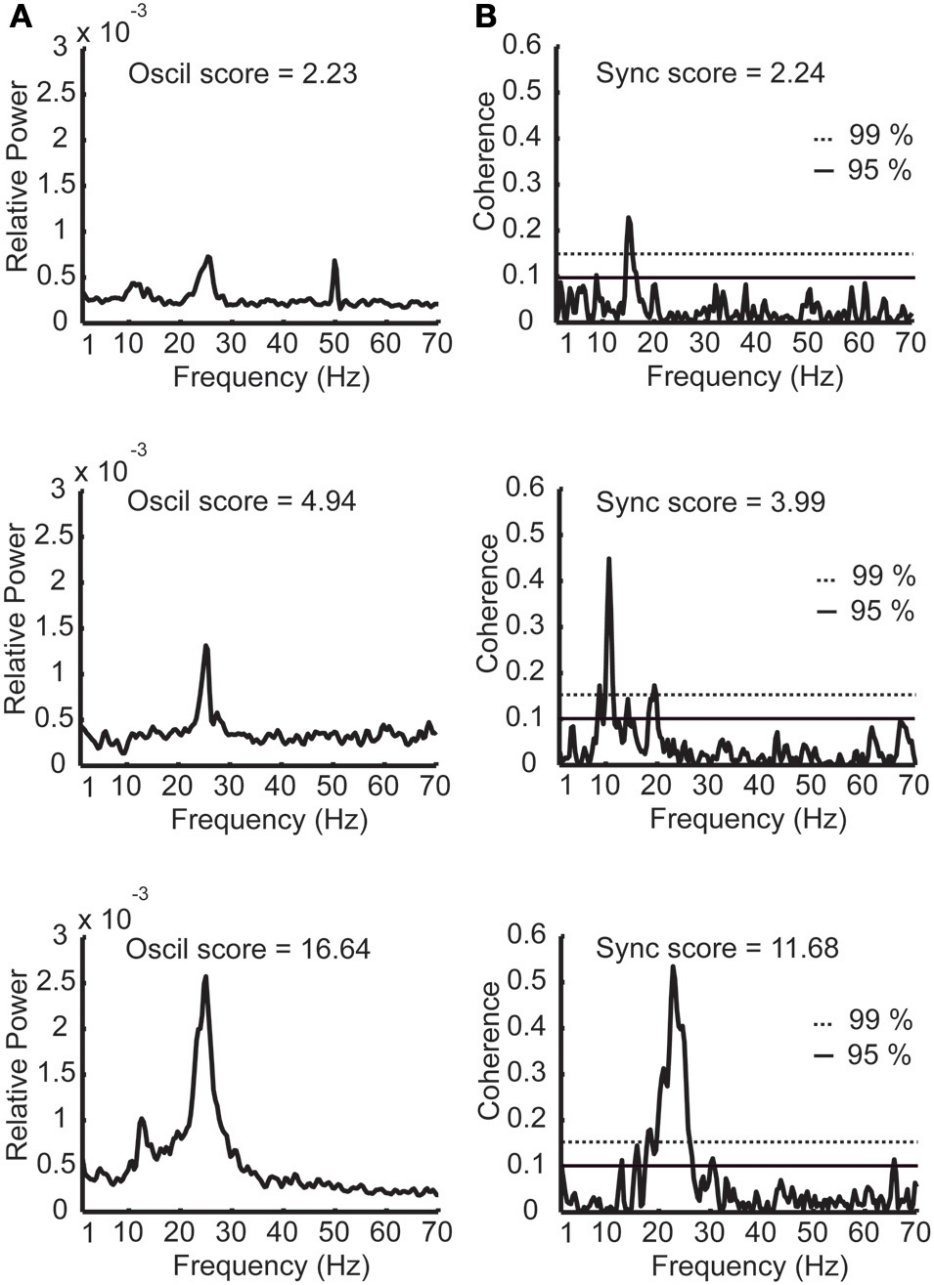
**Examples of power spectra and coherence functions with different oscillation and synchronization scores. (A)** Three examples of power spectra with low, medium, and high oscillation (*oscil*) scores. **(B)** Three examples of STN coherence functions with low, medium, and high synchronization (*sync*) scores. The dashed horizontal and continuous lines denote the confidence interval of 99 and 95% respectively. Sampling duration equals 33.4, 14.5, and 35.1 s for the power spectra examples and 40.2, 41.5, and 40.6 s for the coherence examples (yielding similar confidence intervals for the coherence functions).

Rosenberg and Halliday (Halliday et al., 1995, 2000; Farmer et al., 1997; Farmer, 1998) proposed a very useful method to estimate coherence significance. However, this method employs a threshold confidence level, and does not offer a quantitative measure of synchronization strength. Therefore, a *Z*-score like method (effective *Z*-score: Z^*^) was devised to determine the synchronization and oscillation strength. The *Z*-score of a given parameter is defined as the number of standard deviations above (or below) the mean. In this case the parameter was the maximum value (peak value) of the smoothed PSD or coherence (see below). However, instead of using the standard deviation of the entire frequency range, a *tail* standard deviation (σ_tail_) was defined in the frequency range of 35 to 70 Hz. In this range, no coherence or power spectrum phenomena were observed in our dataset (Figures 1D, 2A–C, 3A,B, 4B). To smooth the coherence, a simple moving average (SMA) was calculated, with a window size of 23 samples (7.67 Hz), and a delta of one sample (i.e., the frequency resolution of 1/3 Hz). The synchronization strength or score was defined as Z^*^ = (MAX(SMA(C (*f*))) − μ/σ_tail_). MAX(SMA (C (f))) is the maximum value of the coherence after smoothing with the moving average, and μ is the coherence mean. To find the frequency in which the spectrum or the coherence achieved maximal value, the arg-max(SMA(*C*(*f*))) was calculated. The coherence (*C*(*f*)) maximal peaks were defined in the smoothed coherence function with minimal distances of 5 Hz between them. The search for the coherence peak was started at the lower frequency, and progressed to the largest value of the smoothed coherence function. All calculations (max, mean and arg-max) were performed in the frequency range between 1 and 70 Hz. Negative scores were found in a few cases, due to residual high power at the low (1–2 Hz) frequency range, and these were ignored.

To determine the oscillation strength, the same effective *Z*-score as for the synchronization was used and defined as the *oscil score*. The maximum value of the smoothed PSD (by a simple moving average, with window size of 23 samples, and delta of one sample, i.e., 1/3 Hz), and the *tail* standard deviation (σ_tail_) in the frequency range of 35–70 Hz were calculated. The *oscil score* was defined as Z^*^ = (MAX(SMA(PSD (f))) − μ/σ_tail_). MAX(SMA(PSD (f))) is the maximum value of the PSD after smoothing with the moving average; μ is the PSD mean.

To explore the relationship between oscillation and synchronization a statistical measure of the oscillation strength of the two oscillatory sites was used. The average PSD^*^ = (PSD1 + PSD2)/2 was calculated, where PSD1 and PSD2 were the power spectrum densities of each site in the neuronal pair, and the *oscil score* of PSD^*^ was calculated. Additionally, other estimates of *oscil scores* of the two sites were calculated as: *min*(*oscil*_1_, *oscil*_2_); *max*(*oscil*_1_, *oscil*_2_); and as the geometric mean of the two scores where *oscil*_1_, *oscil*_2_ were the *oscil scores* of each PSD site. The geometric mean was calculated as: oscil = sign(*oscil*1 * *oscil*2) * GeoMean(|*oscil*1|, |*oscil*2|), where *sign* was the sign operator of *oscil*_1_ and *oscil*_2_ product, in the case of negative values.

Synchronization or oscillations were defined to be significant when the scores reached the Z^*^ ≥ 2 (i.e., the coherence or the PSD peak value was higher than 2 *SD* of the mean values of these functions).

### Coherence confidence level

To assess the validation of *sync score* the confidence level (CL) of the coherence analysis (Halliday et al., 1995) was used. We divided the microelectrode records of duration R into L non-overlapped disjoint segments of duration S (R = L^*^S). The total spectrum was calculated using the average of the magnitude-squared (MS) of the discrete Fourier transform (periodogram), after removing the local mean in each segment S. Each segment contained S = 2^∧^16 samples with a frequency resolution of 0.7336 Hz. Only complete segments were analyzed; data points at the end of the record that did not make a complete segment were not included in the analysis. The procedures were implemented using Neurospec free MATLAB toolbox: http://www.neurospec.org. To obtain the approximate confidence interval for 95% and 99% from the data points, the level thresholds: CL_95_ = 1 − 0.05^1/(*L* − 1)^ and CL_99_ = 1 − 0.01^1/(*L* − 1)^, respectively, were used. Figure 4B depicts examples of the relations between MS-coherence estimates (*Z*-scores) we used in the manuscript with coherence confidence levels of 95% and 99% respectively.

### Assessing the causal relations between oscillations and synchrony

Spurious synchronization can arise from non-coupled oscillatory sites that oscillate in the same frequency bands (i.e., two atomic clocks might be synchronized due to their exact frequency although there is no physical coupling between them (Strogatz, 2003). To rule out this spurious oscillation-synchronization, the mean coherence of randomly shuffled pairs (10,000 times) was calculated for each category (all pairs, DLOR-DLOR, VMNR-VMNR, and DLOR-VMNR) of the STN. The shuffling was performed using the Mersenne Twister algorithm (Matsumoto and Nishimura, 1998) with a different seed number in each iteration.

## Results

### Synchronization occurs only between DLOR pairs

Figure 1 show an example of synchronous oscillatory activity as recorded by two electrodes inserted into the STN of a PD patient during DBS procedures. The raw analog data is shown in two time scales in Figures 1A,B. The power spectrums and the coherence function of this recording are shown in Figures 1C,D, respectively. One can easily observe the synchronous oscillations in the beta range (~20 Hz) in this example.

To explore the properties of STN neuronal synchronization, STN spiking activity simultaneously recorded from two electrodes was analyzed (Figure 2). In total, 2390 multi-unit pairs along 72 STN trajectories (with >4 mm STN span in both electrodes) from 57 PD patients undergoing DBS surgery were included in the analysis.

Previous physiological studies of the basal ganglia in the rodent (Mallet et al., 2008a,b) and primate (Bergman et al., 1994; Nini et al., 1995; Raz et al., 1996, 2000, 2001; Goldberg et al., 2002) models of PD have indicated abnormal synchronicity of basal ganglia neurons as one of the major changes occurring in the network following dopamine depletion. Nevertheless, when the neuronal synchronization of simultaneously recorded STN sites (over the entire STN 2390 pairs) was measured, no distinguishable synchronization was found.

The STN can be spatially differentiated into sub-regions according to neural activity (Zaidel et al., 2010). Two areas could be robustly discriminated in our recording: the dorsolateral oscillatory region (DLOR, *n* = 1778 sites, Figure 3A) and the ventromedial non-oscillatory region (VMNR, *n* = 3002 sites, Figure 3B). Figures 3C,D show the distribution of the *oscil scores* in the DLOR and VMNR, respectively. As expected, significantly higher *oscil score* values were observed in the DLOR than in the VMNR.

The division of the STN into the DLOR and VMNR domains enabled testing of the synchronization of STN pairs from the same and different regions. Significant synchronization, mainly in the frequency band of 8–30 Hz was found, but only between pairs in the DLOR itself (DLOR-DLOR, *n* = 615 pairs, Figure 2A, upper subplot). This synchronization was not observed in pairs of electrodes at DLOR and VMNR (*n* = 548 pairs, Figure 2B, upper subplot) or in the VMNR (*n* = 1227 pairs, Figure 2C, upper subplot). This finding is consistent with previous multiple electrode studies of the human Parkinsonian STN (Levy et al., 2000, 2002a,b; Amirnovin et al., 2004; Weinberger et al., 2006, 2009; Alavi et al., 2013; Lourens et al., 2013) which reported coherence between STN oscillations in a small fraction of STN pairs. However, our findings indicated that the topographical location of the STN electrodes affected the probability of finding a correlation between STN sites, and coherence was only and robustly found between DLOR-DLOR multi-unit pairs.

Recent imaging studies (Lambert et al., 2012; Haynes and Haber, 2013) have clarified that the boundaries between the functional subdomains of the STN are fuzzy, and an overlap of motor and non-motor projections can be found in the transition areas between the STN domains. Therefore, the average coherence at the dorsolateral and ventromedial STN was tested with increasing gaps (0.5–2 mm) from the HMM borders. These results are shown in the lower five rows of Figure 2, and reveal a sharpening and increase of the average coherence peak in the STN DLOR when the gap is increased. Similarly, when the *oscillation scores* are calculated along the normalized depth of the DLOR, a gradual decrease in the oscillatory scores is observed as the DLOR lower border is approached (Figure 2D).

The above results were obtained by averaging over pairs recorded for different durations. The average coherence results (Figure 2) was further compared to the average coherence results of the same pairs with homogenous intervals (only the first 10 s of each recording was included, and recordings with durations shorter than 10s were excluded). Similar results (data not shown) were obtained.

### Synchronization vs. oscillations in the DLOR area

Next, correlation between the oscillations and synchronization in the STN was analyzed. The oscillation and synchronization strengths were calculated using the *oscil* and *sync scores* for each pair in the DLOR area. Figures 4A,B depict three examples of power spectrum and coherence function of STN activity with their relative *oscil* and *sync scores*, respectively. See also Figure 1 for an example of a simultaneous recording of two sites in the STN, and their corresponding values of *oscil* and *sync scores*.

**Figure 5:**
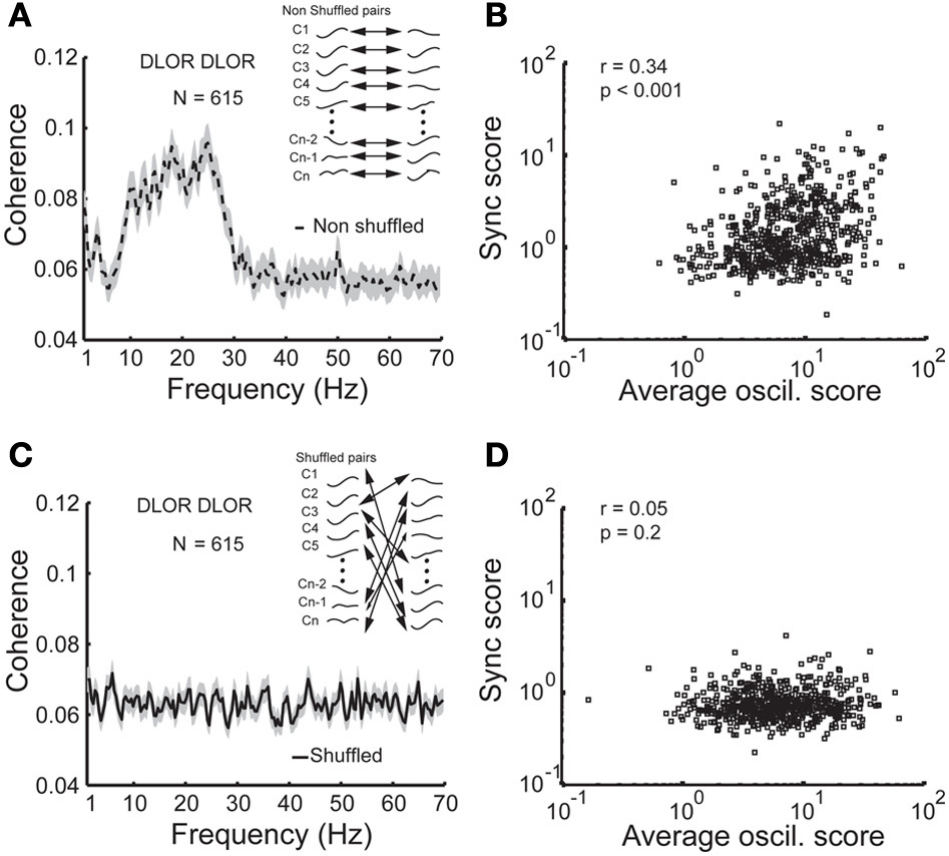
**Synchronization is no longer apparent between non-simultaneously recorded (shuffled) STN DLOR pairs. (A)** A significant average coherence between non-shuffled pairs within the STN DLOR. *N* = 615 represents the number of DLOR-DLOR pairs. **(B)** Scatter plot of synchronization *(sync)* and oscillation *(oscil)* scores in the STN DLOR reveals that the two measures are correlated. Each square represents the synchronization (*Y*-axis) vs. average (arithmetic mean) of the two oscillation scores of one of the 615 pairs within the DLOR. *r* is the Spearman correlation coefficient and *p* is the probability that *r* = 0 (no correlation between the scores). **(C)** Synchronization is no longer seen between non-simultaneously recorded (shuffled) STN DLOR-DLOR pairs. Inset: Schematic illustration of the shuffling procedure. The shuffling procedure was repeated 10,000 times for each pair. **(D)** Scatter plot of shuffled oscil-sync score values. Same conventions as in **(B)**.

Figure 5B depicts the scatter plot of the *oscil* and *sync scores* for all DLOR pairs. Different indicators for the oscillation strength of pairs of STN sites were used: the minimal and maximal *oscil score*, the arithmetic average of the PSDs, and the geometric mean of *oscil scores*. In all cases, the scatter plot of the *sync score* vs. the *oscil score* of the pairs within the DLOR area (*n* = 615) indicated a significant correlation (*r* > 0.24, *p* < 0.001) between the synchronization and the oscillations. Here (Figure 5B) we show only the data for the arithmetic mean of the *oscil. score*s.

**Figure 6:**
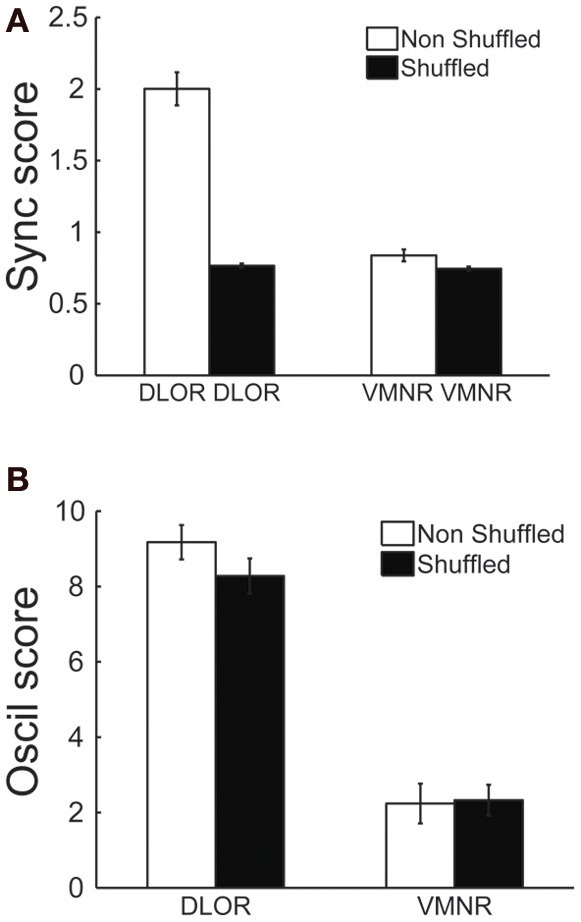
**Average values of synchronization (*sync score*) in the DLOR, but not average VMNR synchronization or oscillations scores in both STN domains, are affected by the shuffling procedures. (A)** Synchronization (*Sync*) scores before (white) and after (black) shuffling, lines indicate standard error of the mean (SEM). **(B)** Oscillation (*Oscil*) scores before and after shuffling. Same conventions as in **(A)**.

The correlation between the oscillation and synchronization strength could imply that the synchronization pattern was dependent on the homogeneity of the neuronal oscillations within the DLOR. If the neural oscillations in different sites of the STN of a single patient have a very stable and equal frequency, the existence of synchronization may not be the result of physical coupling between the STN neurons. Therefore, the DLOR pairs of each trajectory were randomly shuffled and the synchronization between the shuffled (non-simultaneously recorded) pairs was re-quantified in each trajectory. After shuffling, the oscillations remained in the same frequency band, but the synchronization was no longer apparent. Figures 5A,C show the average coherence functions before and after shuffling of the DLOR-DLOR pairs (*n* = 615), respectively. Figures 6A,B depict the average *sync* and *oscil scores* before and after shuffling in the STN DLOR and the VMNR. As expected, shuffling had no significant effect on the *oscil score* in either area (oscillation is a property of a single element and therefore should not be affected by the shuffling procedure). However, the average *sync score* of the DLOR pairs, but not the VMNR pairs declined significantly after the shuffling procedure (Figure 6A). Finally, Figure 5D depicts the scatter plot of the *sync* and *oscil scores* of the shuffled pairs within the DLOR. The Spearman correlation between the *sync score* and *oscill score* dropped dramatically from r1 = 0.34 to r2 = 0.05 (*p* < 0.001 for the null assumption that r1=r2). The mean PSD estimate for the average *oscil score* (as in Figure 5B) was used for this analysis. Similar results were obtained for the other indicators of oscillation strength of the STN pairs.

### Coherence is mainly in the beta frequency range

**Figure 7:**
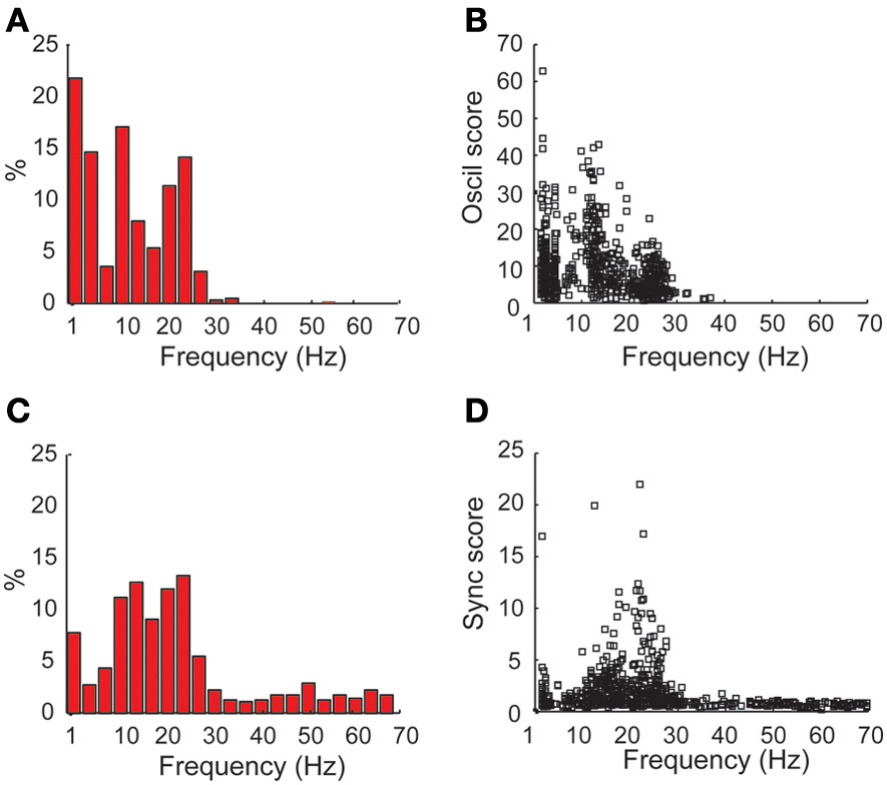
**Dominance of coherence at the beta frequency range vs. tremor frequency and beta oscillations in the power spectrums in the STN DLOR. (A)** Frequency distribution of the frequencies with maximal power in the power spectrums of STN DLOR recording sites. **(B)** Scatter plot of oscillations (*oscil*) scores vs. the frequency with maximal power. **(C,D)** show the frequency distribution and scatter plot, respectively, for the coherence, same conventions as in **(A,B)**. *N* = 1230 single DLOR sites and 615 DLOR-DLOR pairs.

Next, the frequency value where each spectrum (Figure 7A) and coherence (Figure 7C) reached its maximal value was calculated. In both cases, a bi-modal distribution was observed, with a dominance of tremor frequency (3–7 Hz) and beta (12–30 Hz) oscillations, for the auto-spectrums and the coherence functions, respectively. Figures 7B,D show the scatterplot of the maximal *oscil* and *sync scores*, respectively, as a function of their frequency. While the *oscil scores* had similar values in the beta and the tremor range, the values of the maximal *sync scores* were much higher in the beta than in the tremor range. These results are in line with our previous primate studies (Raz et al., 2000) that revealed mainly 5 Hz peaks in the auto-correlations vs. higher frequencies (10 Hz) in the cross-correlations functions of pallidal units and pairs recorded in the globus pallidus after MPTP treatment. However, we cannot rule out the possibility that the 10 Hz activity in this study is not tremor related and a harmonic feature (or n:m locking) of the tremor or of the neuronal oscillations at the tremor frequency.

### Lack of positive correlation between the STN *oscil* and *sync scores* vs. PD symptoms

Previous studies have suggested that STN oscillations and synchronization are correlated with tremor in PD patients (Levy et al., 2002a). This would indicate that the STN synchronized oscillations are driven by the tremor (which may be generated by an independent neuronal loop). The above findings of robust synchronization in the beta rather than in the tremor frequency range (Figure 7D) are not in line with this hypothesis. Nevertheless, we looked for correlations between the *oscil* and *sync scores* of our patients and their pre-operative (OFF medication) UPDRS scores. We did not find significant positive correlation between the average *oscil score* and *sync scores* of STN activity and the UPDRS scores of the tremor in the contra-lateral upper limb(s), all tremor (including axial) scores, and all UPDRS III motor scores. There is a trend for STN synchronized beta oscillations to be more robust in patients with less tremor. While these results might point to a correlation between STN beta oscillations and akinetic/rigid Parkinsonian symptoms (an issue that requires clarification in future studies with bigger sample of patients and with intra-operative clinical assessment), they definitely indicate that the STN beta synchronized oscillations are not a by-product of the PD tremor.

## Discussion

In this manuscript, synchronization within the human Parkinsonian subthalamic nucleus was investigated. No significant synchronization was found over the STN as a whole. After dividing the STN into two electro-physiologically distinct regions, the dorsolateral oscillatory region (DLOR) and the ventromedial non-oscillatory region (VMNR), significant synchronization in the beta range was observed, however, only within the DLOR. The strength of the DLOR synchronization was correlated with the strength of the oscillations of the multi-unit pairs. Nevertheless, shuffling between DLOR pairs abolished synchronization, suggesting that STN synchronization is an independent phenomenon and not a mere reflection of neuronal oscillations at similar frequencies.

Previous studies have shown significant spatial overlap between the DLOR and the STN sensorimotor area (Rodriguez-Oroz et al., 2001; Zaidel et al., 2010). The finding that the STN VMNR (considered to be part of the limbic and associative basal ganglia network) remains unsynchronized is consistent with the predominantly motor nature of PD. However, the (normal) lack of synchronization in the STN VMNR may be due to a selection bias of our DBS patients. Since conventional inclusion criteria were used to select candidates for DBS, patients were usually severely motor-impaired and had few of the non-motor features of the disease. Furthermore, the DLOR may reflect the pathological area of the STN which progressively invades the limbic domains of the STN as the disease advances. Finally, our results are in line with a fuzzy rather than a sharp boundary between the STN sub-domains (Lambert et al., 2012; Haynes and Haber, 2013).

### The STN spiking population activity is synchronized

In this study population spiking (multi-unit) activity was used as a measure of the spiking activity of the STN rather than the more classical parameter of single unit activity (Perkel et al., 1967; Abeles, 1982; Lemon, 1984; Eggermont, 1990). This was primarily for practical reasons. The goal of physiological recording in the operating room (OR) is to enable better identification of the borders of the subthalamic nucleus and its sub-regions. The electrode is therefore advanced in 100 μm steps rather than 2–5 μm steps as is customary in the research laboratory setup. The sampling duration at each step is also limited (Shamir et al., 2012) and the OR conditions often do not allow stable recordings (as compared to 30–90 min stable recordings in a research setting). On the other hand, the cross-correlation of composite spike trains derived from several un-discriminated cells recorded on a single electrode (multi-unit activity) enhances the sensitivity of correlation methods. First, the higher discharge rate of multi vs. single unit recording reduces the asymmetric sensitivity of correlation methods to excitation vs. inhibition (Aertsen and Gerstein, 1985). Second, multi-unit cross-correlation can be a more sensitive detector of a neuronal relationship than single-unit cross-correlation (Bedenbaugh and Gerstein, 1997). Thus, use of a multi-unit signal is warranted for both practical and theoretical reasons. Furthermore, the use of signals recorded by two different electrodes in this study reveal the long range (2 mm) synchronization of STN DLOR. It is hoped that future studies of STN units using objective metrics for quantification of the quality of the unit isolation (Joshua et al., 2007; Hill et al., 2011) will shed more light on synchronization in the STN and other basal ganglia structures of human patients.

### Synchronization only occurs between DLOR pairs in the STN

Early studies described neuronal synchronization in the STN as an epiphenomenon found mainly in patients presenting with tremor (Levy et al., 2000, 2002a). More recent studies (Hanson et al., 2012; Alavi et al., 2013; Lourens et al., 2013) have reported that synchronization can be found between some but not all STN pairs. On the other hand, beta-band LFP and spike oscillations have been described as a consistent feature of human PD in the dopamine depleted state (Brown and Williams, 2005; Foffani et al., 2005; Little et al., 2012). Moreover, many studies have documented the consistency of beta-band oscillations in both the spatial and temporal domains (Bronte-Stewart et al., 2009; de-Solages et al., 2010; Zaidel et al., 2010; Abosch et al., 2012; Little et al., 2012). In this study, synchronization within the Parkinsonian STN DLOR was indeed found to correlate with oscillations. However, the shuffling procedure revealed that STN synchronization was not due to independent oscillators with a similar oscillation frequency (Strogatz, 2003). If this had been the case, a significant synchronization should also have been observed between the shuffled (non-simultaneously recorded pairs of the same patient) DLOR-DLOR pairs. Thus, the synchronization of the simultaneously recorded STN pairs probably reflects the increased coupling between these neurons in the dopamine depleted state of Parkinson's disease. This increased coupling is probably due to the increased efficacy of the common inputs to the STN cells, either from the cortex (Nambu, 2004; Kita and Kita, 2012) or from the external segment of the globus pallidus (Plenz and Kitai, 1999; Tachibana et al., 2011). However, at this stage the possibility of increased coupling by lateral connectivity within the STN cannot be ruled out (Parent et al., 2000; Parent and Parent, 2007).

The finding that most of the energy of the STN synchronous oscillations is in the beta range suggest that these oscillations are not generated by feedback of the peripheral tremor. It is interesting to note that synchronous oscillations in the basal ganglia of MPTP treated primates are mainly found in the 10 Hz domain, where human oscillations span the full beta range (12–30 Hz). Future studies should reveal if this is due to species difference, or due to differences between the MPTP model and human idiopathic Parkinson's disease.

## Concluding notes

In this study we show that the STN domain most affected by PD dopamine depletion (the DLOR, probably the STN motor domain) exhibited both oscillations and synchronization. This suggests that synchronization reflects an additional property of the Parkinsonian STN. Previous studies in the basal ganglia of MPTP treated primates have demonstrated that synchronization can be completely independent of oscillatory activity (Heimer et al., 2002). The previous and the current findings can serve to recast the relationship between oscillations and synchronization in the Parkinsonian basal ganglia (Raz et al., 2000; Amirnovin et al., 2004; Moran et al., 2008). In addition to changes in discharge rate and pattern, synchronization within the STN may be another pathophysiological marker of Parkinson's disease. The potential consequences of synchronization (as opposed to other attributes like rate and pattern change) are probably mainly due to reduced information capacity of the basal ganglia neurons. However, the different pathological changes in the parkinsonian basal ganglia are probably not mutually exclusive. Synchronized oscillations have stronger effects than less synchronized oscillations and completely unsynchronized oscillations might have no effect on target neurons. Furthermore, future studies toward adaptive DBS (Rosin et al., 2011) should investigate which of the pathophysiological changes in the STN activity might be used as the optimal trigger for closed loop DBS.

## Conflict of interest statement

The authors declare that the research was conducted in the absence of any commercial or financial relationships that could be construed as a potential conflict of interest.
